# Monolithic Perovskite–Silicon Dual‐Band Photodetector for Efficient Spectral Light Discrimination

**DOI:** 10.1002/advs.202308840

**Published:** 2024-03-09

**Authors:** Woochul Kim, Yeonju Seo, Dante Ahn, In Soo Kim, Chandran Balamurugan, Gun Young Jung, Sooncheol Kwon, Hyeonghun Kim, Yusin Pak

**Affiliations:** ^1^ Sensor System Research Center Korea Institute of Science and Technology (KIST) Seoul 02792 Republic of Korea; ^2^ Ceramic Total Solution Center Korea Institute of Ceramic Engineering and Technology Icheon Gyeonggi 17303 Republic of Korea; ^3^ Nanophotonics Research Center Korea Institute of Science and Technology (KIST) Seoul 02792 Republic of Korea; ^4^ Department of Energy and Materials Engineering Dongguk University‐Seoul Seoul 04620 Republic of Korea; ^5^ School of Materials Science and Engineering Gwangju Institute of Science and Technology Gwangju 61005 Republic of Korea

**Keywords:** dual‐band photodiode, monolithic stacking, perovskite, selective spectral discrimination

## Abstract

Selective spectral discrimination of visible and near‐infrared light, which accurately distinguishes different light wavelengths, holds considerable promise in various fields, such as automobiles, defense, and environmental monitoring. However, conventional imaging technologies suffer from various issues, including insufficient spatial optimization, low definition, and optical loss. Herein, a groundbreaking advancement is demonstrated in the form of a dual‐band photodiode with distinct near‐infrared‐ and visible‐light discrimination obtained via simple voltage control. The approach involves the monolithic stacking integration of methylammonium lead iodide (MAPbI_3_) and Si semiconductors, resulting in a p‐Si/n‐phenyl‐C_61_‐butyric acid methyl ester/i‐MAPbI_3_/p‐spiro‐MeOTAD (PNIP) device. Remarkably, the PNIP configuration can independently detect the visible and near‐infrared regions without traditional optical filters under a voltage range of 3 to −3 V. In addition, an imaging system for a prototype autonomous vehicle confirms the capability of the device to separate visible and near‐infrared light via an electrical bias and practicality of this mechanism. Therefore, this study pushes the boundaries of image sensor development and sets the stage for fabricating compact and power‐efficient photonic devices with superior performance and diverse functionality.

## Graphical abstract

A practical perovskite‐silicon dual‐band photodetector based on a back‐to‐back photodiode is designed that can discriminate the wavelength region for photoresponse through simple voltage control without the use of optical filters. The proposed single‐pixel imaging platform is capable of real‐time identification of mixed VIS and NIR light.

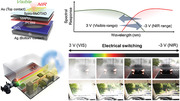

## 1 Introduction

The selective detection and identification of light wavelengths provides abundant and detailed visual information to improve our perception, understanding, and interaction with the world. This technology extends beyond human vision and drives innovation and advances in sophisticated machine vision, automotive image sensors, biomedical imaging, environmental and security monitoring, and military and defense applications.^[^ ^1^, ^2^, ^3^, ^4^, ^5^ ^]^ Specifically, distinguishing or extracting visible (VIS, wavelength (λ) = 400–750 nm) and near‐infrared (NIR, λ = 750–1400 nm) information in real‐time is critical to enhancing the visibility and accuracy of automatic vision systems to achieve facile object recognition, hazard identification, and situation judgment.^[^ ^6^, ^7^ ^]^

Currently, the predominant approach for achieving wavelength selectivity employs broadband Si photodiodes coupled with optical cutoff filters (CFs).^[^ ^8^, ^9^ ^]^ For example, NIR‐CFs, which block wavelength bands above 750 nm, can effectively mimic human color perception patterns by mitigating the color distortion caused by infrared light; this is known as redshift imaging.^[^ ^10^, ^11^ ^]^ Conversely, VIS‐CFs integrated with Si or Ge diodes have demonstrated utility in tracking NIR light reflected from objects in dark or foggy environments, where such light might be imperceptible.^[^ ^12^, ^13^ ^]^ However, the use of optical CFs is intrinsically associated with significant light loss (1/3 of the incoming light), hindering their practical use in low‐light environments.^[^ ^14^ ^]^ Furthermore, different viewing angles with respect to the individual bands in filter array‐equipped devices require additional image processing and error correction, resulting in excessive power consumption and slow imaging process.^[^ ^15^, ^16^, ^17^ ^]^

Dual‐band photodiodes based on new materials, such as transition metal chalcogenides, perovskites, and oxide semiconductors, enable high photon‐to‐charge conversion efficiency and carrier mobility characteristics, thereby attracting research attention for addressing the dramatic increase in information throughput.^[^ ^18^, ^19^, ^20^ ^]^ Heterojunction devices, including MoS_2_/Ge,^[^ ^21^ ^]^ MAPbBr_3_/nonfullerene acceptor (CO*_i_* 8DFIC),^[^ ^22^ ^]^ and ZnO/MoS_2_,^[^ ^23^ ^]^ have been reported and characterized by large‐band offsets at the conduction or valence band interfaces. However, the spectral controllability of these devices has not yet reached a sufficient level to replace filter‐coupled Si photodiodes.

Recently, significant efforts have been made to develop back‐to‐back photodiode (BPD) structures consisting of two diodes configured in PNP or PINIP arrangements to enhance the optical responsiveness and spectral discrimination efficiency.^[^ ^24^, ^25^, ^26^ ^]^ The primary feature of BPDs is their capacity to selectively activate a single diode under an applied bias. Dual‐band BPDs have been successfully engineered using diverse cutting‐edge materials, including Si, Ge, organic compounds, 2D materials, and quantum dots.^[^ ^27^, ^28^, ^29^, ^30^, ^31^, ^32^ ^]^ Although a wide range of studies have demonstrated spectral discrimination based on BPD, material combinations to split the VIS and NIR spectra based on a 750 nm cutoff point, which is the key for realizing practical image sensing, are yet to be reported.

In this study, we introduced a groundbreaking dual‐band photodiode technology that can select the wavelength region for the photoresponse through simple voltage control. This simple approach and suitable material combination enables the separation of the VIS and NIR regions based on wavelength range of 700–800 nm without optical CFs or post‐image processing. We employed two distinct semiconductors, namely methylammonium lead iodide (MAPbI_3_) with a bandgap of 1.6 eV for the VIS light absorption and Si with a bandgap of 1.12 eV for the NIR light absorption, to obtain this photodiode. These materials were integrated into a PNIP BPD configuration of (p‐type Si/n‐type phenyl‐C_61_‐butyric acid methyl ester (PCBM)/intrinsic MAPbI_3_/p‐type spiro‐MeOTAD) BPD configuration. Positive and negative biases facilitated the extraction of photogenerated carriers from the MAPbI_3_ and Si diodes, respectively, resulting in identifiable spectral responses to VIS and NIR illumination. The novel BPD exhibited good efficiency for VIS (<750 nm) and NIR (>750 nm) light imaging with minimal distortion (rejection ratio of 10^3^), facilitating the identification of objects under light scattering. Moreover, a single‐pixel imaging platform based on BPD for dual‐band detection was successfully used to probe the capability for the real‐time identification of mixed VIS and NIR light. Therefore, our work provides significant advancements in photodiode technology with promising applications in mobility and vehicular technologies.

## 2 Results and Discussion

**Figure 1:**
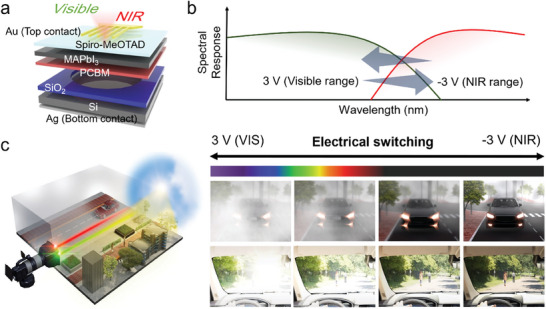
a) Device structure of the BPD. b) Bias‐switchable spectral response of BPD. c) Schematics of the BPD‐based vision system under a foggy environment with excessive white light.

We designed a novel BPD based on Si (bandgap of 1.12 eV^[^ ^33^ ^]^) and MAPbI_3_ perovskite (bandgap of 1.6 eV, Figure S1, Supporting Information) as photoabsorbers to detect NIR and VIS light, respectively, as shown in **Figure** **1**a. An n‐type PCBM was intercalated between the two layers and served as an electron transport layer. A p‐type spiro‐MeOTAD organic semiconductor was used as a hole‐transport layer to promote hole extraction from the MAPbI_3_ layer.

The PNIP structure, i.e., Si/PCBM/MAPbI_3_/Spiro‐MeOTAD, exhibits an efficient energy band diagram, in which each diode is electrically configured in the opposite direction (Figure S2, Supporting Information). Therefore, the photoresponse at certain wavelengths in the VIS region can be adjusted to the NIR region by applying an electrical bias, as shown in Figure 1b. The tunable spectral response can be a key feature of an optical component for the development of imaging hardware vision systems that can be deployed in autonomous vehicles, as shown in the schematic in Figure 1c. In low‐visibility situations, such as a foggy weather, the NIR‐CF‐equipped sensors have a limited ability to identify objects. In other cases, transient bright light due to excessive VIS light causes flares, which is unavoidable in conventional Si image sensors, regardless of the NIR‐CFs. However, a BPD‐based vision system, whose spectral response depends on an external bias, can selectively extract NIR information relevant to the target object under ambient VIS light noise, thereby realizing a flare‐free vision system.

**Figure 2:**
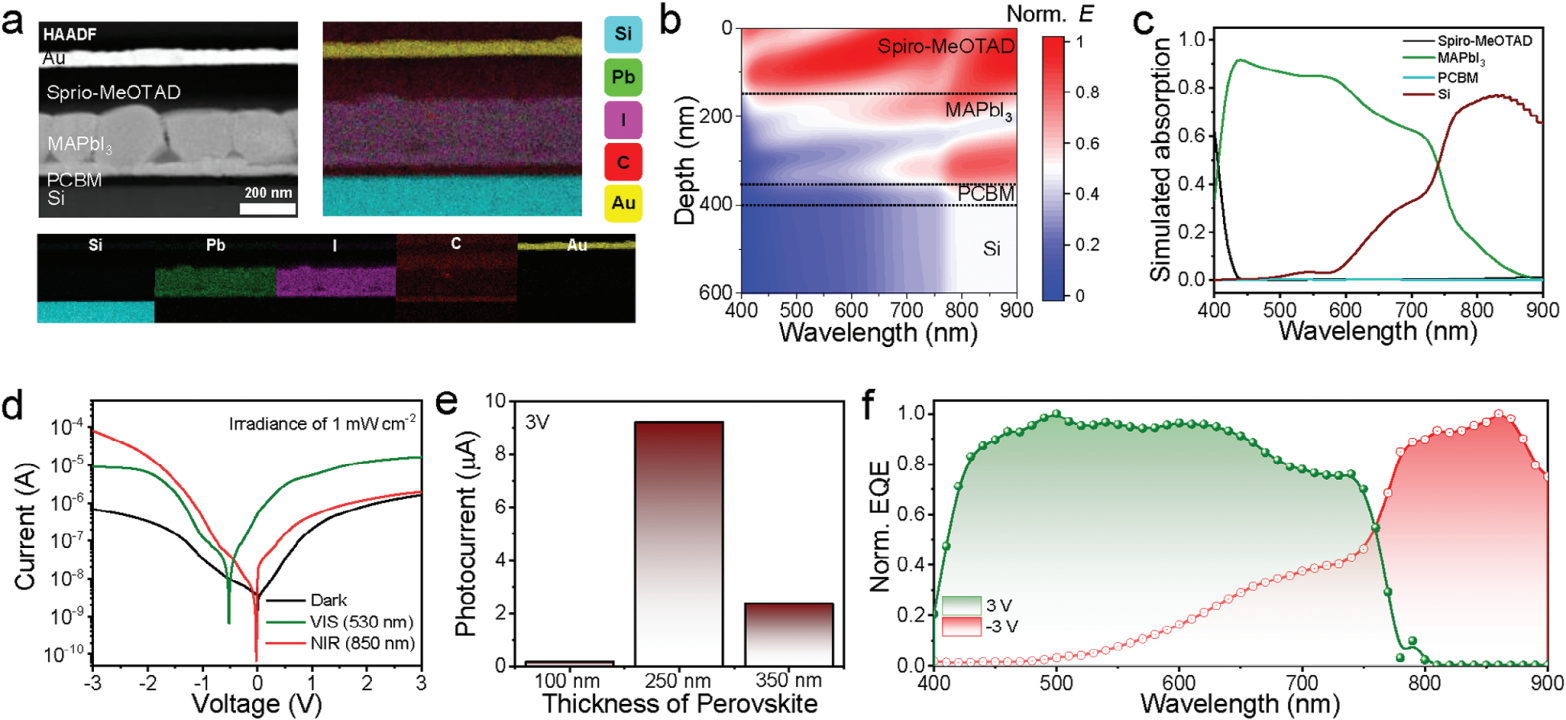
a) HAADF–STEM image of the BPD device and its corresponding elemental mapping images for Si, Pb, I, C, and Au. b) Simulation of the optical field (/E/) profile within the full device configuration under the illumination of a monochromatic light. c) Simulation of the optical absorption of Si, PCBM, MAPbI_3_, and spiro‐OMeTAD layers. d) *I*–*V* characteristics of the BPD under dark and 530‐ and 850‐nm illuminated conditions (the irradiance of LEDs was commonly fixed at 1 mW cm^−2^). e) Photocurrents of the BPD device depending on the thickness of the MAPbI_3_ layers. f) Spectral response (EQE) normalized to the maximum values at 500 and 860 nm at 3 and −3 V, respectively.

**Figure** **2**a shows a cross‐sectional image of the back‐to‐back perovskite films analyzed using high‐angle annular dark‐field (HAADF) scanning transmission electron microscopy (STEM). Layers of PCBM (≈30 nm), MAPbI_3_ (≈250 nm), spiro‐MeOTAD (≈150 nm), and Au (≈50 nm) were uniformly stacked onto the Si substrate (350 µm). A 150 nm‐thick of Ag film was deposited on the bottom side of the substrate. Perovskite thin films were formed by PbI_2_ pre‐vaporization and sequential solution‐based MAPbI_3_ conversion. This method is effective for protecting the underlying organic layer (i.e., PCBM), which is vulnerable to polar aprotic solvents, such as dimethylformamide or dimethyl sulfoxide.^[^ ^34^ ^]^ Elemental analysis using energy‐dispersive X‐ray spectroscopy revealed the composition and distribution of each layer. Pb and I are uniformly distributed within the MAPbI_3_ layer and perfectly separated from Si by PCBM, confirming the absence of the physical degradation of PCBM during fabrication. The highly enriched carbon distributions at the top and bottom of the MAPbI_3_ layer represent spiro‐MeOTAD and PCBM, respectively.

Optical field simulations were performed under monochromatic irradiation (400–900 nm; Figure 2b) to investigate the theoretical absorption within the BPD. The simulations were performed based on transfer matrices,^[^ ^35^ ^]^ and the required complex refractive indices of the MAPbI_3_, Si, spiro‐MeOTAD, and PCBM layers as a function of wavelength were investigated using ellipsometry (Figure S3, Supporting Information). The illumination direction is configured to be incident from spiro‐MeOTAD toward the Si substrate. In the event of an opposite direction, the Si substrate absorbs all incident light due to the thickness of the layer, exceeding the penetration depth of VIS/NIR lights. This leads to a voltage‐independent behavior, which is not conducive to our primary objective. Considering the optical field distribution, incident light with a wavelength of <750 nm was mostly absorbed by MAPbI_3_, whereas incident light with a wavelength of >750 nm could reach the Si layer. Figure 2c shows the theoretical light absorption of each layer based on the optical field simulation, indicating the different light absorption characteristics of each layer, that is, the strong VIS light absorption of MAPbI_3_ and low and intense NIR light absorption of the Si layer. Furthermore, the absorption spectra were simulated by varying the MAPbI_3_ thickness, as shown in Figure S4 (Supporting Information). The absorption curve in the MAPbI_3_ layer gradually flattened across the entire VIS region and that in the Si layer became NIR‐selective with increasing MAPbI_3_ thickness, supporting the filtering effect of the perovskite, which separated the light absorption zone.

Figure 2d shows the photocurrent as a function of the voltage measured by irradiating the BPD (250‐nm thick MAPbI_3_) with 530 and 850 nm light beams. An open‐circuit voltage of ≈−0.5 V was only recorded under 530 nm light irradiation, indicating photovoltaic charge generation at a relatively low bias only in the perovskite diode. In contrast, no shift was observed under the 850 nm illumination for all fabricated devices. A The voltage offset difference of ≈0.5 V is mainly attributed to the difference in the width of the depletion regions between MAPbI_3_ and Si that has ≈10^12^ and ≈10^17^ cm^−3^ of carrier concentration, respectively.^[^ ^26^ ^]^ The photocurrents at the negative and positive voltages demonstrated NIR‐ and VIS‐selective responses, respectively. At −3 V, the photocurrent due to NIR light is dominant, and the photocurrent due to VIS light is mostly negligible. In contrast, at 3 V, the photocurrent under the VIS light considerably increased and overwhelmed the photocurrent generated by NIR light. The generation and transport properties of photogenerated carriers determine the photocurrent intensity; hence, optimizing the thickness of the light‐absorbing layer is essential. As such, we experimentally optimized the film thickness to 250 nm to maximize the inversion characteristics of the photocurrent at 3 V (Figure 2e; Figure S5, Supporting Information). Furthermore, considering that the blocking capability of MAPbI_3_ in the 500–750 nm range of visible light becomes saturated beyond 200 nm, a thickness of 250 nm is still effective in terms of NIR suppression (Figure S4, Supporting Information).

The external quantum efficiency (EQE) of the optimized 250 nm‐thick perovskite device was analyzed as a function of the monochromatic wavelength of 400–900 nm (Figure 2f; Figure S6, Supporting Information). The spectral curves were normalized to the maximum external EQE recorded for each bias. EQE spectra similar to the theoretical absorption profile (Figure 2c) were obtained. The highest detectivity values of the BPD were recorded as 7.7 × 10^11^ Jones (2.5 A W^−1^ under 530 nm illumination) and 8.9 × 10^12^ Jones (30 A W^−1^ under 850 nm illumination) at 3 and −3 V, respectively, as summarized in Figure S7 (Supporting Information). The responsivity above 1 A W^−1^ is normally attributed to a photo‐multiplication (PM) effect associated with trap sites.^[^ ^36^ ^]^ In the device, numerous traps (or defects) exist in each organic or perovskite layers. When a light is illuminated, photo‐generated charge carriers are effectively accumulated at the trap sites interfaces, resulting in an interfacial band bending that facilitates the tunneling injection of charge carriers from the external circuit; thus, an external bias is prerequisite of the PM mechanism. The tendency of inverse proportional relationship between the light intensity and responsivity shown in Figure S7a (Supporting Information) is normally observed in the photodetector operating with the trap‐assisted PM.^[^ ^37^ ^]^ The device exhibited 43 and 92 signal‐to‐noise ratios under weak VIS and NIR light corresponding the 120 and 50 nW irradiance, respectively (Figure S8, Supporting Information).

As shown in Figure S9 (Supporting Information), the BPD maintained the initial photocurrent for 3000 s (5 s on/5 s off) under ambient biases of 3 and −3 V. For long‐term device stability, the device was stored in ambient condition (25 °C and 45% of relative humidity) without any passivation. The photocurrent under VIS light (530 nm) only decreased 4.4% over 2‐week period and that under NIR light (980 nm) reduced ≈5.6% during the same period (Figure S10, Supporting Information). The rejection ratio, defined as the ratio between the photoresponse recorded under illumination at 530 and 850 nm, was higher than 10^3^ at −3 V (Figure S12, Supporting Information), implying that the spectral response range of the BPD clearly varies depending on the engaged bias polarity. Therefore, the photocurrent under the main optical absorption bands (VIS and NIR) of the MAPbI_3_ and Si layers within a single BPD can be independently extracted with minimum band‐to‐band interference depending on the polarity of the applied bias.

**Figure 3:**
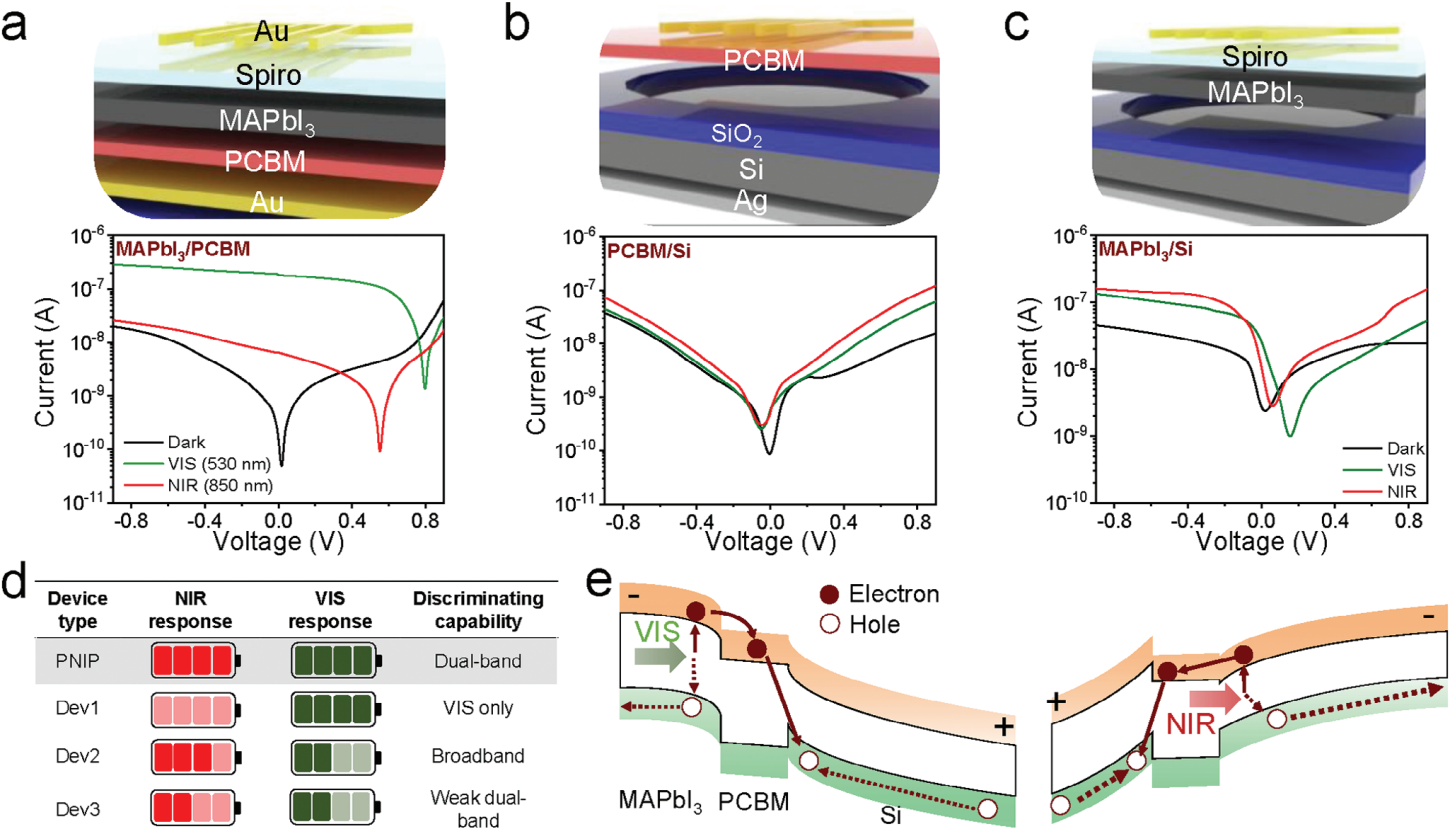
Device structure and *I*–*V* characteristics of a) Au/spiro‐OMeTAD/MAPbI_3_/PCBM/Au, b) Au/PCBM/Si/Au, and c) Au/spiro‐OMeTAD/Si/Au PDs. d) Comparison of the key parameters of the PNIP, *Dev1*, *Dev2*, and *Dev3* devices. e) Energy band diagram of the PNIP configuration in the BPD under the 3 V (left) and −3 V (right) bias.

We prepared three different control groups, namely (1) *Dev1* (NIP MAPbI_3_ photodiode (PD)), (2) *Dev2* (PN Si PD), and (3) *Dev3* (Si/MAPbI_3_ heterojunction PD), to investigate the optical and electrical roles of each component in the MAPbI_3_/Si BPD for dual‐band detection (**Figure** **3**). *Dev1* configured in the NIP diode generated only a photocurrent at the reverse bias (negative voltage), and its photoresponse was highly selective for VIS detection (Figure 3a). *Dev2* has broadband (VIS + NIR) absorption up to the NIR region because of the small bandgap of Si. However, the EQE by VIS and NIR was not dominant, resulting in a small difference in the photoresponse, that is, EQE_NIR/VIS_ ≈1 (Figure 3b). *Dev3*, which is a heterojunction of perovskite and Si, exhibits a stronger photocurrent than *Dev2* in both bias regions (Figure 3c). However, the photocurrent ratio in the VIS and NIR (*I*_VIS_/*I*_NIR_) is less than 3 at 1 V, which is insufficient to realize a clear NIR/VIS distinction. Figure 3d qualitatively and quantitatively summarizes the NIR and VIS photoresponses and discrimination characteristics of the control devices compared with those of BPD.

The energy band diagram in Figure 3e illustrates the principle of transport and extraction of the photogenerated charges in the MAPbI_3_/Si BPD (see the band offset of the heterojunction, Figure S2, Supporting Information). PCBM forms a large valence band offset at the junction with MAPbI_3_ and Si, thereby playing a key role in promoting selective electron transport depending on the applied bias. When multispectral light (VIS and NIR) was irradiated onto the top electrode window, MAPbI_3_ mostly absorbed the VIS light, as shown in Figure 2b,c, and only NIR light reached the Si layer. In Case 1, depicted in the left panel of Figure 3e, the positive bias (positive potential in Si) enforces and weakens the built‐in potential across the MAPbI_3_/PCBM and PCBM/Si interfaces, resulting in the selective extraction of photogenerated charges from MAPbI_3_ associated with the VIS light response. The photoactivated electron, initiated by MAPbI_3_, crosses the PCBM layer via a potential cascade and recombines with the hole in the Si layer to complete the photocurrent flow circuit. In addition, the large valence‐band offset at the PCBM/Si interface, which inhibits hole transport from Si to MAPbI_3_, suppresses charge generation in the Si layer, which interacts with NIR light. Consequently, a selective VIS detection was successfully realized with a considerably high rejection ratio of up to 10^3^ owing to the synergistic effects of the specific voltage polarity and hole‐blocking characteristics of the PCBM. In the other case, the negative bias mainly activates charge generation in the Si layer under NIR light, and the large energy barrier at the MAPbI_3_/PCBM interface inhibits the VIS‐activated hole flux produced in MAPbI_3_, resulting in an NIR‐selective response.

**Figure 4:**
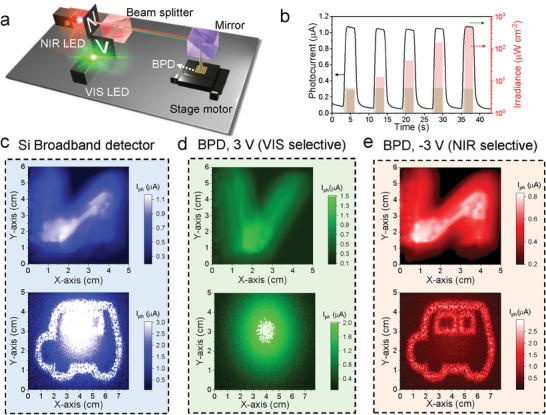
a) Schematic of the optical setup for the VIS and NIR imaging. b) Photocurrent of the BPD at a bias of 3 V under co‐injection of 530‐ and 850‐nm light pulses at 6 µW cm^−2^ with 3 s on and 5 s off from 5 to 500 µW cm^−2^. c) VIS/NIR mixed images of the Si broadband diode (*Dev2* in Figure 3b). d) VIS‐selective images of the BPD at a bias of 3 V. e) NIR‐selective images of the BPD at a bias of −3 V.

We designed a prototype imaging system that can be applied as a driving assistance technology for actual autonomous vehicles to verify the practicality of the photoresponse property for easily and quickly selecting VIS and NIR light with only bias control in a single device (**Figure** **4**a). The information of each character was converged on a beam splitter to deliver mixed character information to the BPD through a mirror using monochromatic light‐emitting diodes at 530 and 850 nm. Selective VIS detection was performed by injecting VIS and NIR light together at 3 V (Figure 4b). We attempted to interfere the device with NIR pulses of 5–500 µW cm^−2^ (3 s on and 5 s off). The photocurrent, which depended on the VIS light intensity (6 µW cm^−2^), was maintained. The linearity of the photocurrent from the BPD was well maintained depending on the light irradiance (Figure S11, Supporting Information), demonstrating the practicality of the BPD‐based system under VIS/NIR light. The impact of VIS light at −3 V was investigated (Figure S13, Supporting Information) under a mixed illumination of a 150 µW cm^−2^ NIR and VIS pulse with different intensities (300–6000 µW cm^−2^). The VIS light with double irradiance resulted in a marginal increase in photocurrent, only 0.5 µA, which is considerably lower than that induced by a single NIR pulse (3.5 µA). Achieving a twofold increase in photocurrent would necessitate elevating the VIS irradiance to 6000 µW cm^−2^, a value 40 times higher than that of the NIR light. In comparison to the high rejection ratio observed at 3 V, the distortion of NIR information, recorded at −3 V, induced by the VIS information may appear substantial. However, when considering a real‐life light condition comprising similar intensities of NIR and VIS lights, the negative voltage condition remains effective in extracting NIR information. Moreover, by combining information recorded under both bias conditions, the light irradiance condition can be estimated more precisely.

Letters “N” and “V” were generated and mixed with the NIR and VIS light, respectively, to validate the performance of the prototype imaging sensor. In addition, the BPD was exposed to appropriate mixed‐light conditions, such as foggy days, overcast days, and strong VIS light from a vehicle in the opposite lane. Figure 4c shows the imaging results obtained using *Dev2* in Figure 3b, corresponding to a commercial Si light sensor (Figure S14, Supporting Information). In the reference Si device, the mixed light information was merged even when the voltage was varied, corresponding to a typical image sensor without a supplementary CF. In contrast, the BPD depicts the selection of NIR and VIS light by voltage control. At a bias of 3 V, only VIS light produced an optical response, producing the selective outputs of the “V” character (top panel in Figure 4d) and headlight flashing light (bottom panel in Figure 4d). In contrast, when switched to a bias of −3 V, NIR information can be extracted to clearly display the letter “N” and vehicle images (Figure 4e). Generally, a dual‐band image can be easily distorted by band‐to‐band interference and misregistration,^[^ ^38^ ^]^ requiring a high‐performance computing algorithm for imaging correction. In this respect, BPD has the advantages in improving band discrimination performance with a large rejection ratio (>10^3^), decreasing the error due to a difference in viewing angle.

## 3 Conclusion

In this study, we successfully developed a novel BPD using Si and MAPbI_3_ perovskites as photoabsorbers for detecting NIR and VIS light, respectively. Through careful design and engineering of the PNIP (Si/PCBM/MAPbI_3_/Spiro‐MeOTAD) structure, the BPD demonstrated excellent dual‐band photoresponse capability. We selectively enhanced or reduced the photoresponse to specific wavelengths within the VIS and NIR spectra by applying an opposite electrical bias. This remarkable feature holds considerable promise for imaging hardware vision systems, particularly for applications in autonomous vehicles, where visibility and detection accuracy are the paramount for safety. The successful performance of the BPD‐based prototype imaging technology is attributed to the excellent photoresponse properties achieved through the optimized junction structures and careful material selection. Further, we leveraged the strengths of each component to control the photogenerated carrier activation and achieve a high photocurrent and excellent rejection ratio in dual‐band detection by exploring the complex interactions of the PCBM, MAPbI_3_, and Si layers. Rigorous experiments and theoretical simulations demonstrated subtle control of the optical absorption bands, which has not been previously reported in a single‐stacked thin‐film device. These results represent an important advancement in optical sensing and imaging applications. Moreover, they open new opportunities for developing ultrasmall photonic devices and sensor technologies with excellent discrimination performance, minimal power consumption, and superior performance without supplementary CFs.

## 4 Experimental Section

### Fabrication of the MAPbI_3_/Si PD

For the device fabrication, a Si wafer with the 300 nm of SiO_2_ layer (<100>, p‐type, 1–10 Ω · cm, iTASCO) was pre‐cleaned with acetone (DUKSAN), isopropyl alcohol (IPA, DUKSAN), and distilled water (DI) in sequence. A hole pattern (a diameter of 1.5 mm) was made using conventional photolithography consisting of spin‐coating of photoresist (PR), UV exposure, and development. After the developing process, the PR‐coated SiO_2_/Si wafer was immersed in a buffered oxide etchant (Sigma–Aldrich) for 3 min to remove the SiO_2_ layer. The overall photolithography was completed by removing the PR residual on the substrate. The role of the SiO_2_ hole is to precisely define the active area. The grid‐typed electrodes, positioned on the hole region, are collected and connected to a single contact pad. It is important to note that the large pad should be isolated from the Si substrate to suppress the generation of undesired electron–hole pairs, which can lead to an overestimation of device performance. The bare Si surface was then cleaned with DI water. For the surface treatment of the Si surface, the UV ozone treatment was conducted for 20 min. In order to form an electron transport layer, PCBM solution (20 mg mL^−1^ in chlorobenzene) was spin‐coated on the UV ozone‐treated Si surface under a spin speed of 2000 rpm for 30 s. Then, for the preparation of MAPbI_3_ film, the PbI_2_ (Sigma–Aldrich) was thermally deposited on the PCBM layer (0.7 Å s^−1^), and MAI (Sigma–Aldrich) solution with additive (40 mg mL^−1^ in IPA with 0.9 vol % of dimethylformamide) was spin‐coated on the PbI_2_ film at a rate of 4000 rpm for 30 s. After heat treatment of as‐prepared film at 100 °C for 20 min, the MAPbI_3_ conversion process was completed. As a hole transport layer, 0.06 M spiro‐OMeTAD dissolved in chlorobenzene (28.8 µL of 4‐tert‐butylpyridine and 17.5 µL of 1.8 m Li‐TFSI in acetonitrile as additives) was spin‐coated at 2000 rpm for 20 s. Finally, a gold electrode was deposited by electron beam evaporation (0.7 Å s^−1^). The entire process without SiO_2_ etching was performed in a nitrogen‐filled glove box.

### Thin Film Characterization

The cross‐sectional HAADF STEM image of MAPbI_3_/Si BPD was observed using a Tecnai G2 S‐Twin microscope operated at 300 kV. The sample for STEM measurement was prepared by using focused ion beam (FIB) system (Hitachi‐NX5000). Elemental analysis of MAPbI_3_/Si BPD was performed by energy dispersive spectrometer (EDS) mode of STEM with 126.3 eV resolution. The refractive indices of MAPbI_3_, PCBM, and Si for optical field simulation were obtained by using an ellipsometer (Ellipso Technology). The absorbance of the MAPbI_3_ film and Si substrate was measured using a UV–vis spectrometer (Cary 60 UV–vis instrument, Agilent).

### Device Characterization

The IV and spectral response characteristics of the photodetector were measured using a Keithley 4200 source meter under illumination from an LED spotlight (530 and 850 nm, Mightex) and monochromatic light from a Xenon lamp (Sciencetech) equipped with a monochromator (Monora200, Dongwoo optron), respectively. Imaging under VIS and NIR lights was tested by projecting an image mask onto the MAPbI_3_/Si BPD.

## Conflict of Interest

The authors declare no conflict of interest.

## Supporting information

Supporting Information

## Data Availability

The data that support the findings of this study are available from the corresponding author upon reasonable request.

## References

[1] H. Kim , W. Kim , Y. Pak , T. J. Yoo , H. W. Lee , B. H. Lee , S. Kwon , G. Y. Jung , Laser Photon Rev. 2020, 14, 2000305.

[2] L. Mennel , J. Symonowicz , S. Wachter , D. K. Polyushkin , A. J. Molina‐Mendoza , T. Mueller , Nature 2020, 579, 62. 32132692 10.1038/s41586-020-2038-x

[3] T. Zhou , S. Ma , D. Yu , M. Li , T. Hang , Sensors 2020, 20, 1. 10.3390/s20154077 PMC7435453 32707858

[4] J. Wu , Z. Shi , L. Zhu , J. Li , X. Han , M. Xu , S. Hao , Y. Fan , T. Shao , H. Bai , B. Peng , W. Hu , X. Liu , C. Yao , L. Li , W. Huang , Adv. Opt. Mater. 2022, 10, 2102514.

[5] M. Shimoni , R. Haelterman , C. Perneel , IEEE Geosci. Remote Sens. Mag. 2019, 7, 101.

[6] Y. Li , J. Moreau , J. Ibanez‐Guzman , IEEE Trans. Intell. Transport. Syst. 2023, 24, 4716.

[7] M. Brown , S. Süsstrunk , Multi‐Spectral SIFT for Scene Category Recognition , IEEE, Colorado Springs, CO, USA, 2011

[8] X. Zou , Y. Zhang , R. Lin , G. Gong , S. Wang , S. Zhu , Z. Wang , Nat. Commun. 2022, 13, 3288. 35672323 10.1038/s41467-022-31019-7 PMC9174490

[9] Y. Horie , S. Han , J. Y. Lee , J. Kim , Y. Kim , A. Arbabi , C. Shin , L. Shi , E. Arbabi , S. M. Kamali , H. S. Lee , S. Hwang , A. Faraon , Nano Lett. 2017, 17, 3159. 28388090 10.1021/acs.nanolett.7b00636

[10] C. Park , M. G. Kang , Sensors 2016, 16, 719.27213381

[11] IEEE Signal Processing Society., Institute of Electrical and Electronics Engineers, ICIP 2009 : 2009 IEEE Int. Conf. on Image Processing , IEEE, Cairo, Egypt 2009.

[12] M. Al Naboulsi , Optical Engineer. 2004, 43, 319.

[13] X. Zhang , Z. W. Zhang , Sens. Actuators A Phys. 2021, 326, 112713.

[14] T. Sakai , T. Takagi , K. Imamura , K. Mineo , H. Yakushiji , Y. Hashimoto , T. Aotake , Y. Sadamitsu , H. Sato , S. Aihara , ACS Appl. Electron Mater. 2021, 3, 3085.

[15] J. Ma , Y. Ma , C. Li , Information Fusion 2019, 45, 153.

[16] 2015 IEEE Int. Conf. Comput. Photography (ICCP) , Rice University, IEEE, Houston, Texas 2015

[17] N. Zhao , P. B. Catrysse , S. Fan , Adv. Photonics Res. 2021, 2, 2000048.

[18] J. Wang , X. Xu , S. Xiao , Y. Li , W. Qian , J. Yu , K. Zhang , S. Yang , Adv. Opt. Mater. 2021, 9, 2100517.

[19] C. Li , H. Wang , F. Wang , T. Li , M. Xu , H. Wang , Z. Wang , X. Zhan , W. Hu , L. Shen , Light Sci. Appl. 2020, 9, 31. 32194945 10.1038/s41377-020-0264-5 PMC7054320

[20] Y. Liang , C. Xie , C. Y. Dong , X. W. Tong , W. H. Yang , C. Y. Wu , L. B. Luo , J. Mater. Chem. C Mater. 2021, 9, 14897.

[21] A. Hwang , M. Park , Y. Park , Y. Shim , S. Youn , C.‐H. Lee , H. Beom Jeong , H. Young Jeong , J. Chang , K. Lee , G. Yoo , J. Heo , Sci. Adv. 2021, 7, eabj2521. 34910523 10.1126/sciadv.abj2521 PMC8673756

[22] L. Li , H. Chen , Z. Fang , X. Meng , C. Zuo , M. Lv , Y. Tian , Y. Fang , Z. Xiao , C. Shan , Z. Xiao , Z. Jin , G. Shen , L. Shen , L. Ding , Adv. Mater. 2020, 32, 1907257. 10.1002/adma.201907257 32383310

[23] K. L. Kumawat , P. Augustine , D. K. Singh , K. K. Nanda , S. B. Krupanidhi , Phys. Rev. Appl. 2022, 17, 064036.

[24] D. Benedikovic , L. Virot , G. Aubin , J. M. Hartmann , F. Amar , X. L.e Roux , C. Alonso‐Ramos , É. Cassan , D. Marris‐Morini , J. M. Fédéli , F. Boeuf , B. Szelag , L. Vivien , Nanophotonics 2021, 10, 1059.

[25] A. Pelella , A. Grillo , E. Faella , G. Luongo , M. B. Askari , A. Di Bartolomeo , ACS Appl. Mater. Interfaces 2021, 13, 47895. 34581561 10.1021/acsami.1c12050 PMC8517951

[26] W. Kim , H. Kim , T. J. Yoo , J. Y. Lee , J. Y. Jo , B. H. Lee , A. A. Sasikala , G. Y. Jung , Y. Pak , Nat. Commun. 2022, 13, 720. 35132055 10.1038/s41467-022-28374-w PMC8821588

[27] E. T. Simola , A. De Iacovo , J. Frigerio , A. Ballabio , A. Fabbri , G. Isella , L. Colace , Opt. Express 2019, 27, 8529. 31052668 10.1364/OE.27.008529

[28] Z. Lan , Y. Lei , W. K. Edward Chan , S. Chen , D. Luo , F. Zhu , Sci. Adv. 2020, 6, eaaw8065. 32064330 10.1126/sciadv.aaw8065 PMC6994203

[29] Z. Lan , F. Zhu , ACS Nano 2021, 15, 13674. 34319066 10.1021/acsnano.1c04908

[30] P. Wu , L. Ye , L. Tong , P. Wang , Y. Wang , H. Wang , H. Ge , Z. Wang , Y. Gu , K. Zhang , Y. Yu , M. Peng , F. Wang , M. Huang , P. Zhou , W. Hu , Light Sci. Appl. 2022, 11, 6. 34974520 10.1038/s41377-021-00694-4 PMC8720310

[31] T. Y. Chang , P. L. Chen , P. S. Chen , W. Q. Li , J. X. Li , M. Y. He , J. Te Chao , C. H. Ho , C. H. Liu , ACS Appl. Mater. Interfaces 2022, 14, 32665. 35797527 10.1021/acsami.2c06088

[32] X. Tang , M. M. Ackerman , M. Chen , P. Guyot‐Sionnest , Nat. Photonics 2019, 13, 277.

[33] H. Sugimoto , M. Fujii , K. Imakita , S. Hayashi , K. Akamatsu , J. Phys. Chem. C 2013, 117, 11850.

[34] P. Cheng , Y. Li , X. Zhan , Nanotechnol. 2013, 24, 484008. 10.1088/0957-4484/24/48/484008 24196520

[35] G. F. Burkhard , E. T. Hoke , M. D. McGehee , Adv. Mater. 2010, 22, 3293. 20517871 10.1002/adma.201000883

[36] K. Domanski , W. Tress , T. Moehl , M. Saliba , M. K. Nazeeruddin , M. Grätzel , Adv. Funct. Mater. 2015, 25, 6936.

[37] J. Miao , F. Zhang , J. Mater. Chem. C 2019, 7, 1741.

[38] T. Chen , Y. Liu , Remote Sens. 2021, 13, 3351.

