# Supplementary material for: Monolithic Perovskite–Silicon Dual‐Band Photodetector for Efficient Spectral Light Discrimination
Source: Adv Sci (Weinh). 2024 Mar 9;11(21):2308840. doi: 10.1002/advs.202308840 (PMC11151070; doi:10.1002/advs.202308840)

ADVANCED SCIENCE
Open Access

Supporting Information

for *Adv. Sci.*, DOI 10.1002/advs.202308840

Monolithic Perovskite–Silicon Dual-Band Photodetector for Efficient Spectral Light Discrimination

Woochul Kim, Yeonju Seo, Dante Ahn, In Soo Kim, Chandran Balamurugan, Gun Young Jung, Sooncheol Kwon, Hyeonghun Kim* and Yusin Pak**

Supporting Information

Monolithic perovskite–silicon dual-band photodetector for efficient spectral light discrimination

Woochul Kim, Yeonju Seo, Dante Ahn, In Soo Kim, Chandran Balamurugan, Gun Young Jung, Sooncheol Kwon, Hyeonghun Kim*, and Yusin Pak**

Figure S1. Optical bandgap measurement of $MAPbI_3$ layer. a) Absorbance of $MAPbI_3$ (~250 nm) by using UV-Vis spectrometer. b) Tauc plot of $MAPbI_3$ derived from the absorption spectra of Figure S1a. The optical bandgap of $MAPbI_3$ layer was identified as 1.6 eV.

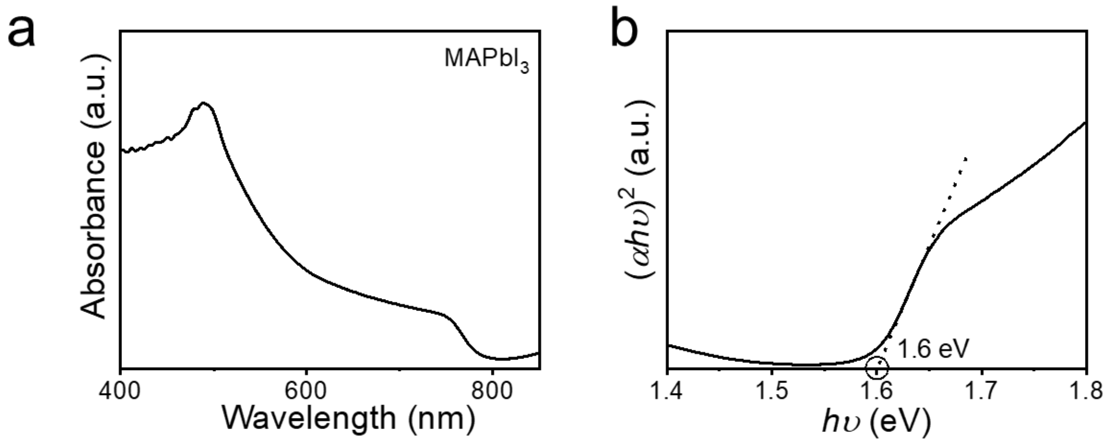

Figure S2. Energy band diagram of the BPD. The energy band diagram of PNI junction in BPD which is consist of Si, PCBM, and MAPbI3 layers. The ΔE_C and ΔE_V are conduction and valence band offset at interfaces, respectively. The E_f and E_g are Fermi level and bandgap of materials, respectively.

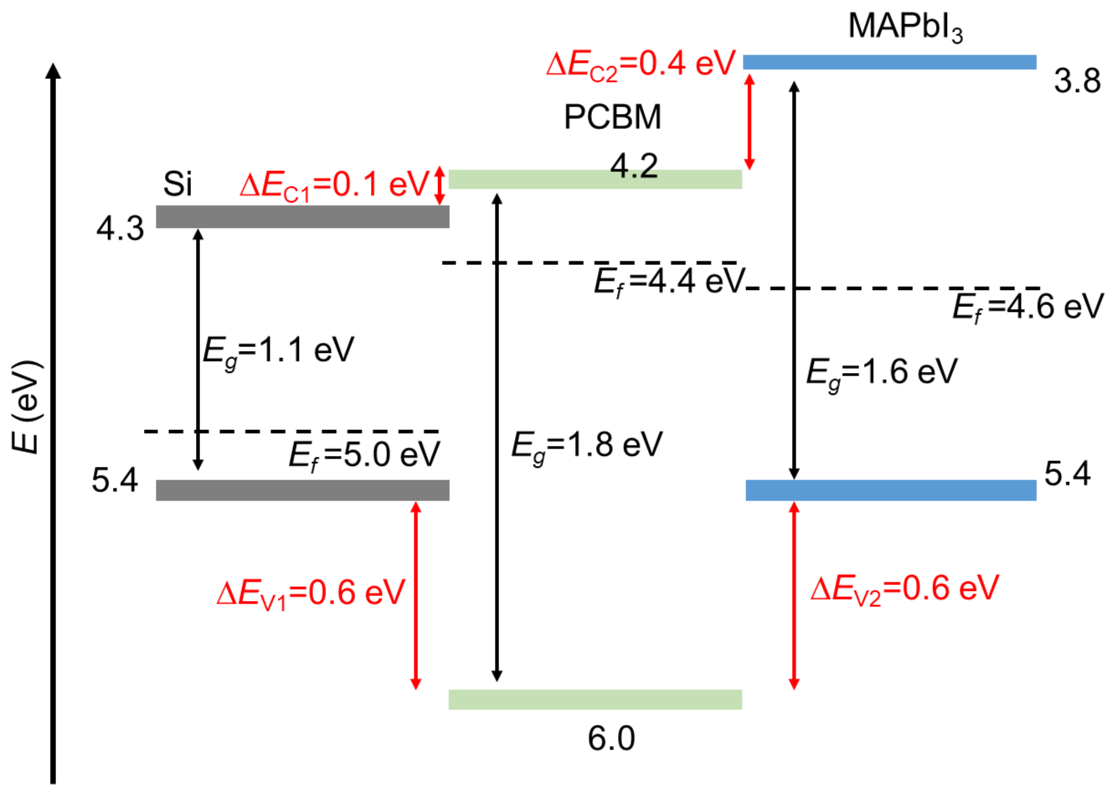

Figure S3. Optical parameters of constitutive layer in BPD for simulating optical field. Complex refractive indices (n+ik) of the a) Spiro-MeOTAD, b) $MAPbI_3$, c) PCBM, and d) Si, were obtained using an ellipsometer.

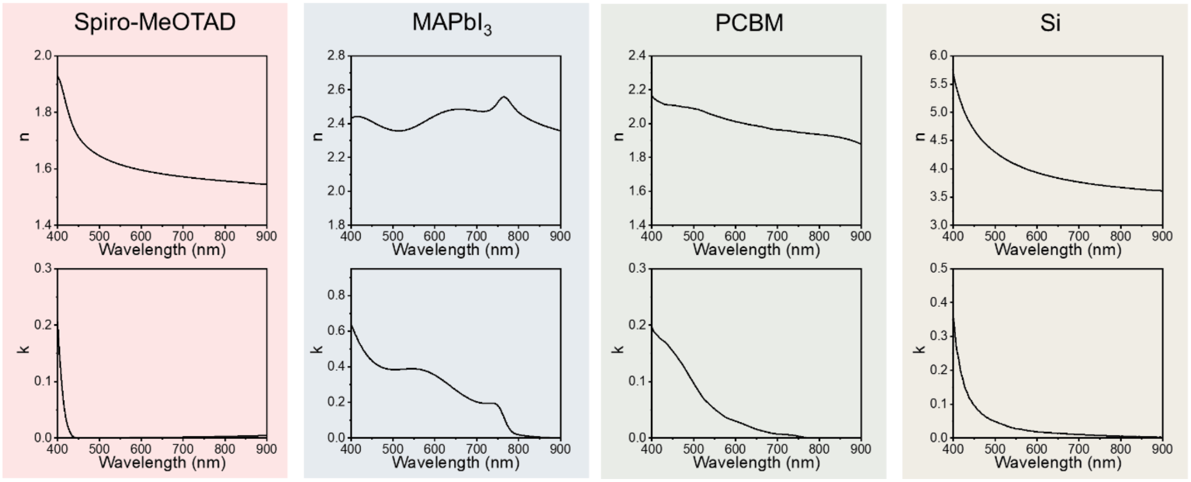

Figure S4. Absorption spectra simulation. Simulated absorbance of $MAPbI_3$ (upper panel) and Si (bottom panel) layer depending on the layer thickness from 0 to 500 nm.

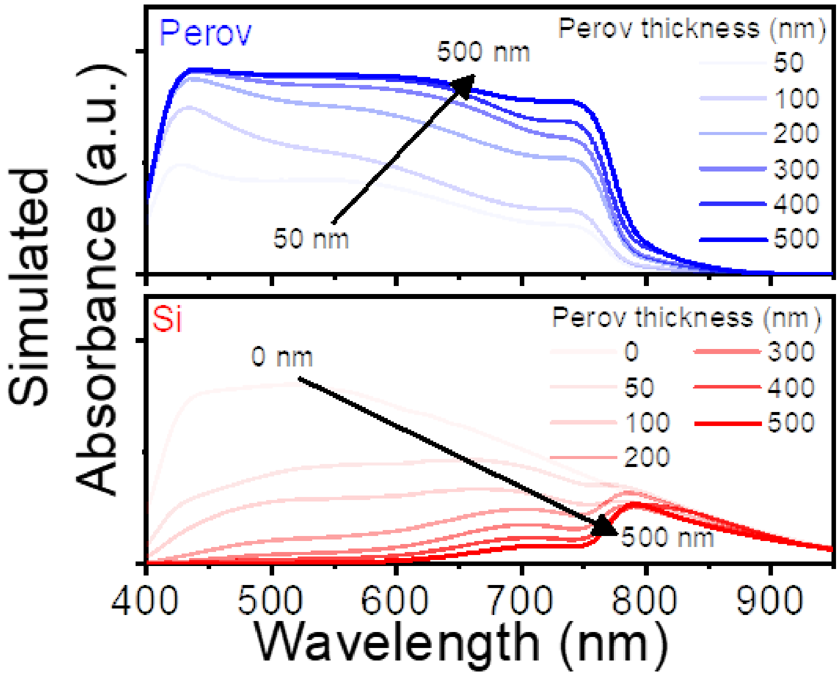

Figure S5. Optimization of $MAPbI_3$ layer thickness. I-V curve of BPDs with 100, 250, and 300 nm $MAPbI_3$ layer under dark and 530 nm illumination.

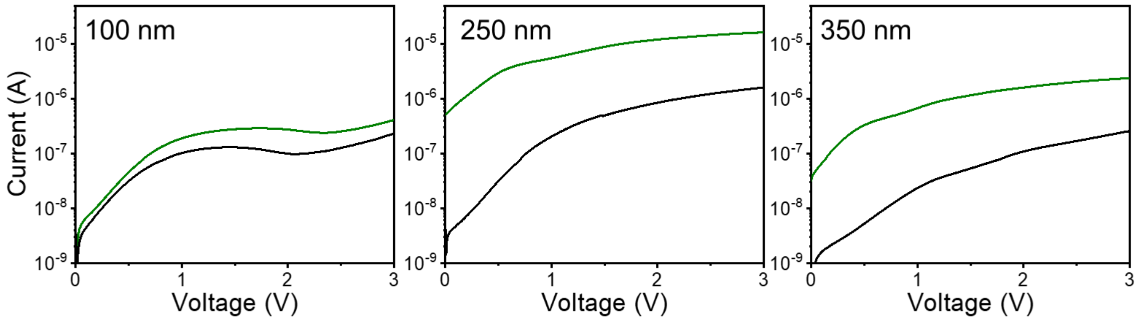

Figure S6. Spectral EQE measurements. a) Light intensity of monochromator-equipped Xenon lamp. Measured photocurrents under the light sources at the voltage of b) -3V and c) 3V. Estimated EQE curves from the light intensity and photocurrent values measured at the

voltage of d) -3 V and e) 3V.

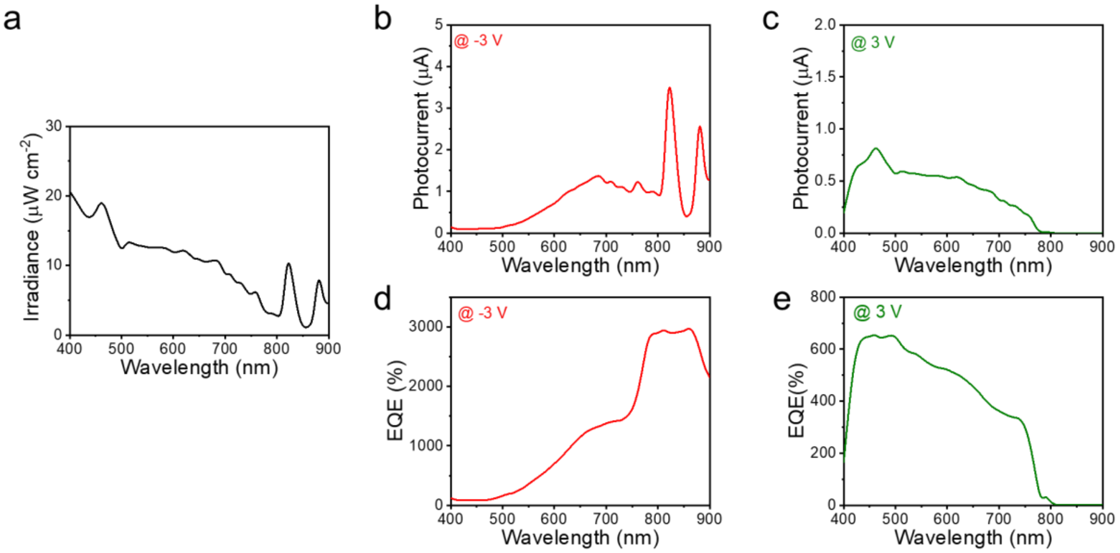

Figure S7. Figure-of-merits of the BPD. a) Responsivity (Rλ) and b) detectivity (Dλ) measured at +3 V and -3 V under illumination of 530 nm and 850 nm (VIS and NIR, respectively) at irradiances from 1 to 10^3 μWcm^{-2}.

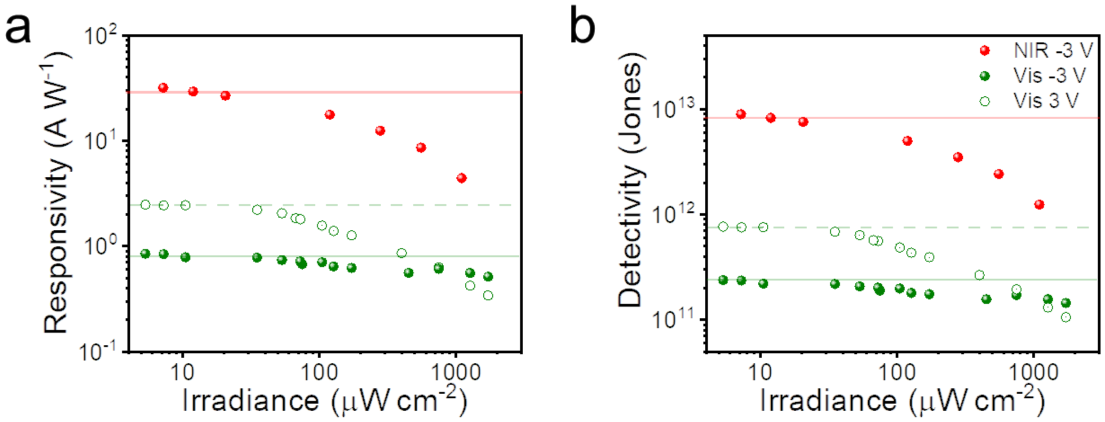

We calculated responsivity (*R*λ) and specific detectivity (*D*λ) as follows (Ref?):

$$R_{\lambda} = \frac{I_{\mathrm{ph}}}{I_{\mathrm{irr}} \cdot A} \tag{S1}$$

$$D_{\lambda} = \frac{R_{\lambda}}{\sqrt{2 \cdot q \cdot I_{\mathrm{d}} \cdot A^{-1}}} \tag{S2}$$

where I_{ph}, I_{irr}, A, I_d and q represent the photocurrent, light irradiance, active area, dark current density, and unit charge, respectively. Equation S2 for estimating the detectivity was employed with an assumption that the dark current noise from the detector is mainly determined by the shot noise.

Figure S8. Dual-band detection performance under low irradiance. Photocurrent measurement of BPD under a) 530 nm illumination of 120 nW irradiance and b) 940 nm light of 50 nW irradiance.

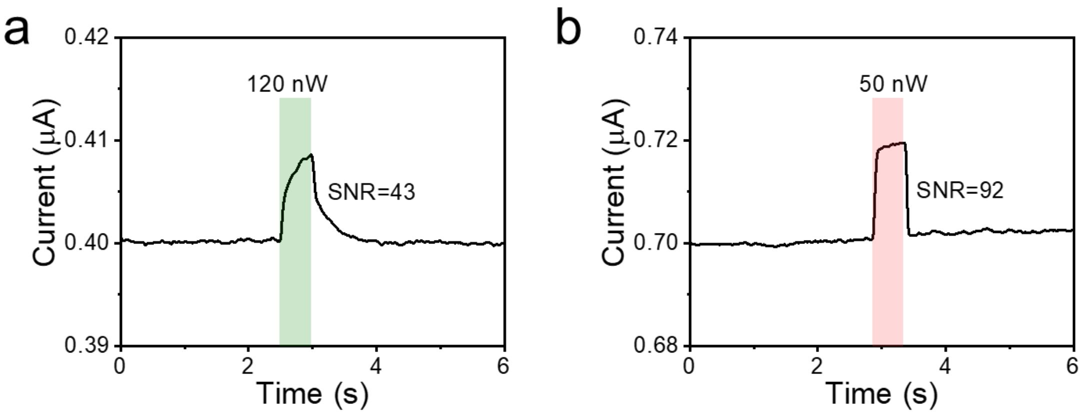

Figure S9. Repeatability test of the BPD. Steady-state photocurrent under light pulse (5 s on/ 5 s off) under illumination of 530 nm at different bias of a) 3V and b) -3V. The measurements were conducted for 3000s under ambient conditions. Left and right inset figures represent the magnified current behavior during 50 s at start and end of measurement, respectively.

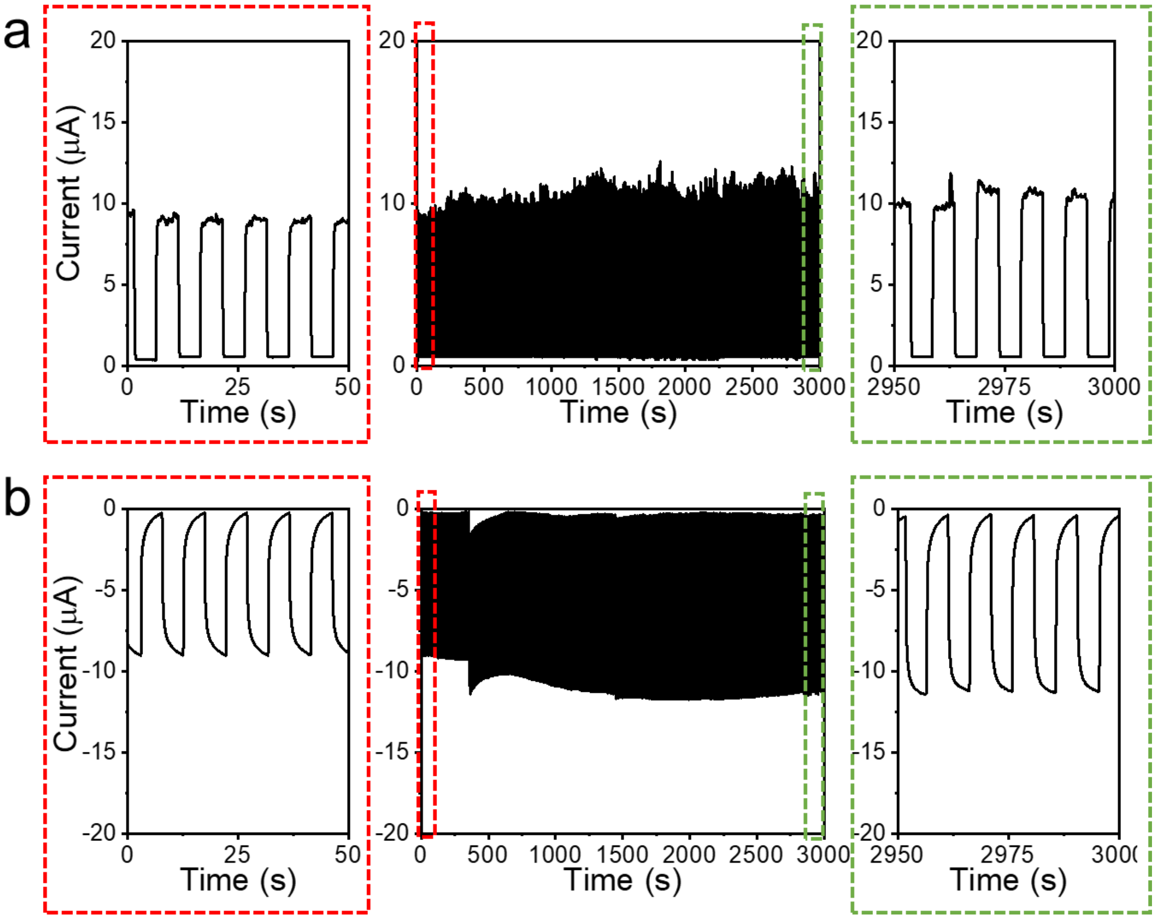

Figure S10. Long-term stability test of the BPD. Photocurrent variation of the BPD under a) 530 nm illumination and b) 980 nm illumination for 2 weeks. The BPD has been stored in ambient condition (25 ºC and 45 % of relative humidity) without any passivation.

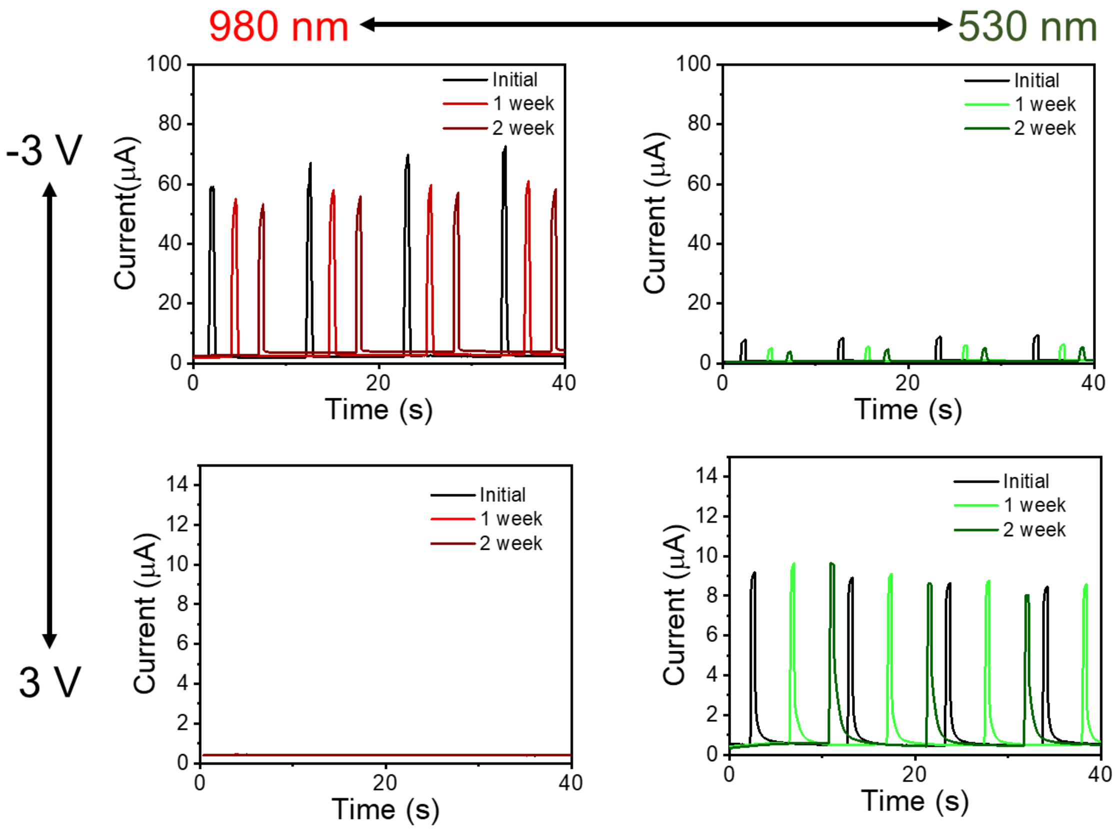

Figure S11. Photocurrent of the BPD depending on the 530 nm light irradiance.

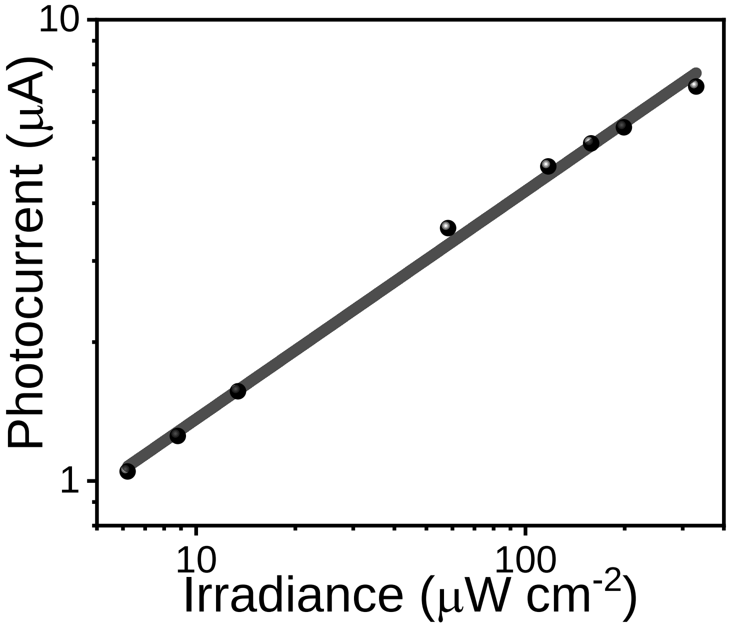

Figure S12. Time-resolved photocurrent measurement under a) VIS (530 nm, irradiance of 80 μW cm^{-2}) and b) NIR (850 nm, irradiance of 1.5 mW cm^{-2}).

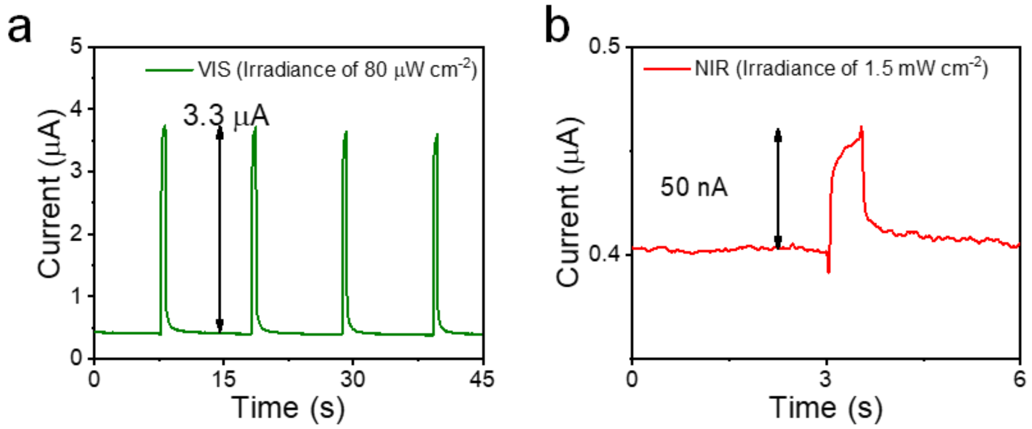

The photocurrent of the device has been incorporated into Figure S12. The photocurrent under visible light (530 nm, irradiance of 80 μW cm^{-2}) is measured at 3.3 μA, corresponding to a responsivity of 2.3 A W^{-1}. In order to observe a measurable current change, the responsivity under near-infrared (NIR) light was assessed with a higher irradiance (1.5 mW cm^{-2}); the resulting photocurrent was 50 nA, and the responsivity was estimated at 1.86 mA W^{-1}. Consequently, based on the responsivity difference, the rejection ratio was calculated as 1240.

Figure S13. Photocurrent of the BPD at a bias of -3 V under co-injection of 530- and 850-nm light pulses.

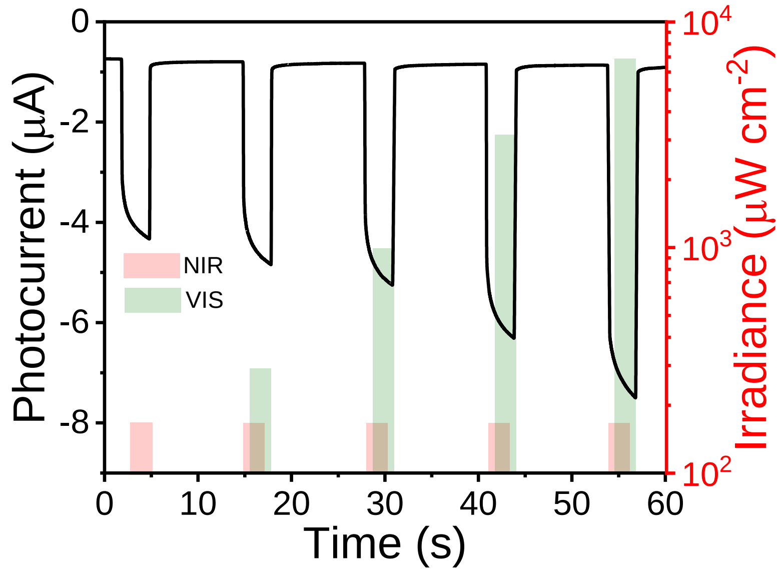

Figure S14. a) Device structure and b) I-V curve of Dev2 under dark, 530 nm, and 850 nm illumination.

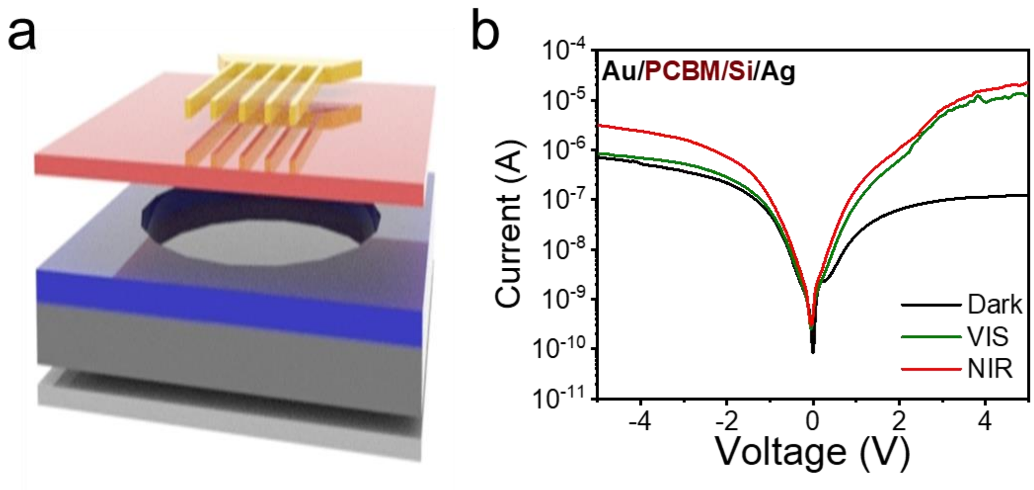

Supplement: Supplementary file 1 — Supporting Information [file ADVS-11-2308840-s001.pdf]
